# The role of caregiver gestures and gesture-related responses of toddlers with autism spectrum disorder

**DOI:** 10.3389/fpsyt.2022.895029

**Published:** 2022-07-22

**Authors:** ShaoLi Lv, Yu Xing, YanTing Xu, LinRu Liu, HuiLin Zhu, QianYing Ye, ChunMei Wang, XiaoBing Zou, HongZhu Deng

**Affiliations:** Child Development and Behavior Center, Third Affiliated Hospital of Sun Yat-sen University, Guangzhou, China

**Keywords:** autism spectrum disorder, caregiver-toddler interaction, caregiver's gesture, social response, synchronization

## Abstract

Autism spectrum disorder (ASD) is characterized by social communicative abnormalities. Deficits and delays in gestural communication are among the early deficits of ASD and also a major social modality in early caregiver-toddler interaction. Caregiver gestures have an important role in the cognitive and social development of children with ASD. Thus, it is urgent to further explore the role of caregiver gestures in early caregiver-toddler interaction. In this cross-sectional study, we observed the caregivers' gestures and responses of toddlers aged between 18 and 24 months during play (ASD = 44, TD = 29) and dining activities (ASD = 34, TD = 27). By observing the different frequencies and patterns of gestures by the caregiver-child interaction and the different proportions of children's responses to the caregiver's gestures, we found that, compared to caregivers of typically developing toddlers, caregivers of toddlers with ASD had fewer synchronized gestures and more unsynchronized gestures in the play activity and more supplementary gestures in dining activity. Toddlers with ASD produced more social responses to caregivers' synchronized gestures, whereas the use of synchronized gestures by the caregivers in caregiver-toddler interaction had a positive influence on social responses to toddlers with ASD. The findings suggest that effective use of gestures by caregivers during caregiver-toddler activities can improve children's social responses.

## Introduction

Autism spectrum disorder (ASD) is a neurodevelopmental disorder defined by social communication deficits and repetitive and restricted behaviors and is induced by the complex interaction between neural susceptibility and behavior (1). An toddlers' behavior is significantly influenced by the social environment, and because the primary early social environment predominantly comprises caregiver-toddler interaction (CTI), CTI may have a significant effect on the onset and development of this disorder (2). In particular, caregiver gestures are an important social modality in CTI and have a positive influence on attracting toddlers' social attention and learning from social experiences, whereas toddlers' social responses influence caregiver gestures to produce adaptive changes, thus influencing CTI in both directions (3). Therefore, an in-depth exploration of the role of caregiver gestures in early childhood social experience may potentially contribute to the early guidance of ASD caregiver-toddler interventions.

Gestures are a stable source of visual information in social communication and provide a positive aid to the development of language comprehension, problem-solving, and subsequent learning skills in socially competent speakers or listeners (4). Different from young adults, gestures can serve as alternative channels of expression to compensate for the limitations of toddlers' language and reflect developmental changes in toddlers' cognitive processes. The gesture input toddlers receive from their caregivers has a ripple effect on their language and social development (3, 5). The use and effects of gestures by caregivers on toddlers with ASD have partially been reported in the past. Caregivers of toddlers with ASD use gestures with similar frequency as caregivers of typically developing (TD) toddlers (6, 7), and some studies have reported that caregivers of toddlers with ASD produce more gestures than caregivers of TD toddlers (3). Additionally, the types of gestures and their combinations with speech have been partially reported. During play activities, the frequency of gesture types (deictic, conventional, and representational gestures) and gesture-speech combinations were similar between caregivers of toddlers with ASD and TD (8, 9). Some studies have reported that mothers of toddlers at heightened risk for ASD use more directive gestures and fewer requesting gestures (10). Studies have also explored the effects of caregivers' gestures on toddlers with ASD language and social skills, including facilitation and prediction of language comprehension and expression (11–13), increasing toddlers' compliance and sharing during play (10).

In summary, the existing literature included investigations of the number of caregiver gestures and contribution of caregiver gestures to children's language development, but few studies have reported the effects of caregiver gestures on children's social responses and social participation in CTI. Further, previous studies have mainly reported on the characteristics and effects of mothers' gestures during play activities, but information on caregivers' gestures during different early family activities is lacking. As for TD children, parental behaviors can promote the development of children with ASD (14). Responsive parenting that follows the child's guidance and focus of attention predicts language and social gains in children with and without developmental risk (15). Additionally, responsive parenting styles predict the total time of child-initiated joint engagement, and children's social behaviors are associated with child-initiated engagement (16). Early quantity, diversity, and grammatical informativeness of verb input in follow-in utterances (i.e., utterances that map onto child attentional leads) are significantly associated with later child expressive verb vocabulary (17). Toddlers and toddlers diagnosed with ASD later often display hyporeactivity to sensory stimuli, which has also been associated with lower child communication abilities, but increased caregiver verbal responsiveness may attenuate this negative effect (18). In the context of spatial activities (puzzle play), caregivers use spatial language and gestures to improve their child's spatial skills, whereas parental spatial talk is related to the child's later spatial skills (19).

Previous studies on CTI have reported the effects of caregivers' behaviors on developmental abilities and disorder symptoms in children with ASD, whereas fewer articles have reported on the responses of children with ASD to their caregivers' behaviors during CTI. Choi et al. investigated gestures that caregivers used with 12-, 18-, and 24-month-old infants at high or low risk for ASD and reported that caregivers of three groups gestured in similar frequencies and proportions (8). However, there is a lack of specific analyses of toddlers' differential reciprocal social responses to caregivers' behaviors.

Several studies have reported the positive effects of increased synchronization (responsiveness to the toddlers' interest and behavior) of CTI on children's social interaction. Caregivers of toddlers with ASD whose behaviors were more responsive to the toddler's ongoing interests and activities during early play interaction would accordingly result in toddlers who developed better joint attention and language (20, 21). At-risk toddlers with higher binary synchrony and toddler reactivity at 12 months achieved significantly higher receptive and expressive language (EL) scores at 36 months (22). The importance of interpersonal synchronization in ASD is supported by studies of motor, physiological, and neural synchrony. For example, reduced levels of repetitive behavior were previously reported in children who demonstrated increased hand motor coherence in hand clapping competitions with the experimenter (23). Additionally, reduced electrical skin, heart rate, and neural synchronizations were reported in binary interaction between ASD and caregivers (24, 25). However, there is a lack of studies on specific synchronous behaviors of caregivers, including synchronous non-verbal and verbal ones.

In summary, this study observed CTI in terms of caregiver gestures and children's specific responses to caregiver gestures. The following hypotheses were proposed in this study: 1. The frequency and use patterns of different gestures by caregiver of ASD differ, and synchronized gesture use by caregiver of ASD may be less; 2. toddlers with ASD may respond differently to caregiver gestures in different activities, and the use of different gesture types by caregivers during play or dining activities may cause toddlers to have different social responses; 3. The use of gestures in caregiver-toddler interaction by caregivers will influence toddlers to produce different social responses. We observed two major caregiver-toddler activities in early childhood, including play activities and daily routines. Further, co-parenting is becoming a common phenomenon in China, and most toddlers and toddlers are raised by at least two caregivers (26). Based on the social context of early childhood in China, and in order to have a more comprehensive picture of the interaction between caregivers and young children, we compared the use of caregiver gestures and toddlers' responses to gestures between the TD and ASD groups by examining dyadic play and dining activities between 18- and 24-month-old toddlers and their two caregivers. Our study questions were as follows:

- Do different types of gestures produce atypical usage patterns among caregivers of children with ASD in different caregiver-toddler activities, including different types of social functions, types of speech combination, and synchronized and unsynchronized gestures?
- Do atypical response patterns of children with ASD to caregiver gestures in different caregiver-toddler activities exist?
- Does a correlation between children with ASD's social response to caregiver gestures and caregiver gestures in dyadic interaction exist?

## Materials and Methods

### Participants

This study was approved by the Ethics Committee of the Third Affiliated Hospital of Sun Yat-Sen University. The ASD group was recruited from the developmental behavior clinical unit and through the program's webpage. The TD group was recruited from the Department of Child Healthcare after health counseling or medical examination. Written informed consent was obtained from all families. Participants consisted of families among which toddlers were classified as TD or ASD groups. Participants in the ASD group met the following inclusion criteria: (a) chronologic age between 18 and 24 months, (b) meeting the Diagnostic and Statistical Manual of Mental Disorders criteria for ASD according to an expert clinician, and (c) the Autism Diagnostic Observation Schedule–Toddler Module (ADOS-T) suggesting that clinical concern was mild to moderate or moderate to severe. A group of chronological age-matched TD children was recruited. The exclusion criteria included the following: serious neurological or physical conditions and diagnosis of Rett syndrome, cerebral palsy, or other congenital disorders. In addition, because toddlers are often shared by two caregivers, to achieve a complete picture of the toddler's early CTI, each child was required to have two caregivers, residing with the child continuously since birth. Since some of the enrolled families did not complete all the videos, we report information only for the families who completed the play and dining videos separately. Demographic information for the TD and ASD groups is provided in Table 1.

**Table 1:** Participant characteristics.

	**Play activities**		**Dining activities**	
	**ASD (*****n*** **= 44)**	**TD (*****n*** **= 29)**	**ASD (*****n*** **= 34)**	**TD (*****n*** **= 27)**
Caregiver characteristics
Caregiver age in years, mean (SD)	36.48 (6.74)	38.16 (7.62)	36.81 (7.05)	37.98 (7.68)
Caregiving time, mean (SD)	5.31 (2.62)	4.93 (2.98)	5.45 (2.66)	4.79 (2.75)
Caregiver education, *n* (%)	16 (36.36)	16 (55.17)	11 (32.35)	13 (48.15)
Household Income, *n* (%)	20 (45.45)	14 (48.28)	19 (55.88)	13 (48.15)
Caregiver, parents, *n* (%)	32 (72.73)	18 (62.07)	24 (70.59)	17 (62.96)
Toddler characteristics
Children age in months, mean (SD)	20.61 (2.30)	20.14 (2.15)	20.85 (2.34)	20.37 (2.26)
Child's sex, boys, *n* (%)	32 (72.73)	14 (48.28)	24 (70.59)	12 (44.44)
*Mullen*, mean (SD)
VR T-score	39.75 (10.72)	54.90 (10.41)	40.32 (10.92)	56.48 (9.93)
FM T-score	42.80 (9.07)	51.34 (6.35)	43.59 (9.54)	52.37 (5.31)
RL T-score	35.52 (15.54)	58.90 (11.06)	36.88 (15.83)	60.04 (10.30)
EL T-score	30.50 (11.07)	42.90 (10.07)	31.41 (12.03)	43.78 (10.05)
ADOS-T CSS	5.91 (1.65)	2.28 (0.88)	5.97 (1.73)	2.26 (0.90)

*Caregiving time indicates the average care time of the two caregivers. Educational qualifications indicate that both caregivers have a bachelor's degree or higher. Parental caregivers indicate that the child's two caregivers are his or her parents. Household income is monthly income of the following: ¥3,000–¥6,500, ¥6,500–¥20,000, ¥20,000–¥30,000, ¥30,000–¥70,000, others, ¥20,000, and above are counted here. Mullen comprises four scales: visual reception, fine motor, receptive language, and expressive language, with the number of toddlers with a T-score > 30 per scale being reported here. ADOS-T CSS, Autism Diagnostic Observation Schedule Calibrated Severity Score*.

### Activities

All children were assessed using the Mullen Scales of Early Learning (MSEL) for non-verbal and verbal abilities (27). Toddlers were evaluated for ASD symptoms using ADOS-T. Notably, although the current recommendation for the average age of diagnosis of ASD is 3 years, however, the previous literature has reported that ASD was independently confirmed at age 3 years in 82.6 and 91.8% of young children diagnosed with ASD at 18 months and 24 months, respectively (28).

**Figure 1:**
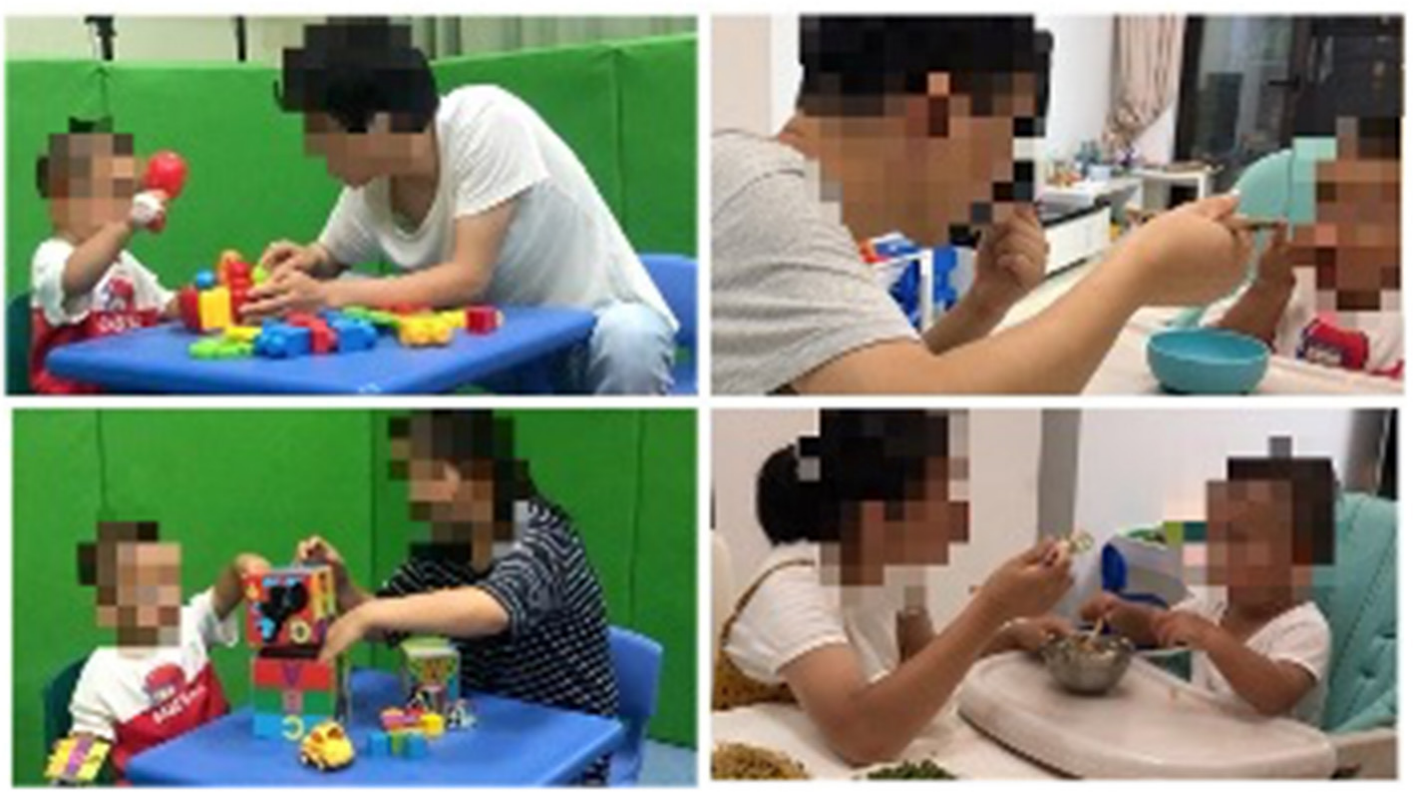
Caregiver-toddler interaction video scene setup and context. Both of the two caregivers complete the video of play and dining activities with their children.

The participating families were instructed to complete four CTI sessions: semi-structured free play activities at the hospital and dining activities at home with two caregivers. All activities were recorded on video for 7 min. The first phase during the first min of the play/dining activities, known as the adaptation phase, allowed the toddlers to adapt to their surrounding environment, the caregiver's placement of them, and adjustment of shooting equipment time. No coding assessment was performed in this phase. Play activities were conducted in a 3 × 2 m^2^ carpeted game room. The room contained a set of toys, including a car, music box, pop-up toy, set of boxes, eight textured blocks, eight building blocks, and eight plastic snowflakes. At the start of each session, we instructed caregivers to select any three toys and then engage in a typical “at-home” play session. Dining activities referred to any daily dining of the toddler by the caregiver in the home setting. The video-taker was instructed to capture the following: (a) the faces of the toddler and caregiver, (b) the hands of the toddler and caregiver, and (c) the toys/utensils used. Background noises (e.g., television, open window) were avoided as much as possible (Figure 1). Play activities were recorded by the researcher and dining activities were recorded by family members who were familiar with the children.

Play and dining activities were selected because of the following reasons: (1) the increasing number of interventions emphasizing the importance of natural situations (29, 30) and early dyadic social activities for toddlers mainly include games and daily routines, of which dining is one of the important and indispensable routines; (2) the two activities cover different patterns of CTI during this period (including differences in behavior and sensory stimulus input patterns on both sides of the interaction and dining activities) based on toddlers' physiological protection mechanisms and requirements for nutritional intake, involve fewer objects, and are usually caregiver-led, whereas play activities include more child behavior patterns and sensory information input from different objects and are usually not caregiver-led; and (3) consider achieving more specific guidance on caregiver gestures in the clinic, structured dining activities may be more helpful in guiding the use of gestures by the caregivers.

### Measures

#### The autism diagnostic observation schedule–toddler module

The ADOS-T is used for toddlers aged 12–30 months and consists of two domains: social affect and restricted repetitive behaviors. Using the algorithm, the following ranges of concerns were identified: little to no concern, mild to moderate concern, and moderate to severe concern (31, 32). The calibrated severity score (CSS) of the ADOS was used to assess the severity of individual modules (33).

#### The mullen scales of early learning: AGS edition

Toddlers' developmental levels were assessed using the MSEL, a measure that comprises four scales: VR, FM, RL, and EL (27). The two non-verbal scales (VR and FM) were averaged to obtain a non-verbal score, and the two verbal scales (RL and EL) were averaged to obtain a verbal score. T-score cutoff values were based on standard deviations and 95% score between 70 and 130, and T-scores <30 on any scale are indicative of significant delay and warrant early intervention.

#### Dyadic interaction coding

The videos of CTI were coded on a per-second basis. All videos entered the coding from the second min of the interaction, and the duration was 6 min. No third-party interaction with caregivers or toddlers other than video were coded. We standardized the manually coded indicators for statistical analysis. The specific treatments were as follows: (1) caregiver gesture frequency, the number of caregiver gestures per 6 min of dyadic interaction (i.e., the total number of caregiver gestures divided by total time and multiplied by 6), and (2) the proportion of toddler's social response, the number of each response type that was divided by the number of all responses recorded in CTI. Video coding of caregiver gestures and toddler responses was performed using ELAN software (34).

#### Caregiver gestures

The caregiver gesture codes included the following: the frequency of gestures, frequency of different types of gestures, different communication functions (35), and different gesture-speech combinations (36). In addition to the above types, we further classified caregiver gestures according to a previously established description of synchronized behavior (20): synchronized gesture, in which the caregiver makes a gesture in synchrony with the object or activity that the toddler is attending to, and unsynchronized gesture, in which the caregiver makes a gesture that is not synchronized with the object or activity the toddler is attending to. The synchronization of caregiver gestures with the toddler's attention included the following: (1) temporal overlap, gestures occurring between the time the toddler initiated and ended attention to the object, and (2) content overlap, gestures toward the same object or thing as the toddler's attention (Table 2).

**Table 2:** Caregiver gestures and types of caregivers.

**Types of caregiver gestures**	**Definition**	**Examples**
**Communication functions**		
Behavior regulation	A gestural act used to manage the behavior of another person.	Pointing to the blocks on the ground and requesting the child to pick it up
Social interaction	A gestural act used to attract or maintain the attention of another for social purposes.	Hand movements to play “peek-a-boo”; waving hi or bye-bye
Joint attention	A gestural act used to direct or share another person's attention to an object or event.	Pointing and looking to the blocks
**Gesture-speech combinations**		
Reinforcing gestures	Gesture conveyed information that was redundant with speech	“Block” + point at block
Disambiguating gestures	Gesture clarified a pronominal, demonstrative or demonstrative referent in speech	“This” + point at block
Supplementary gestures	Gesture added semantic information to the message conveyed in speech	“Yellow” + point at block
**Gesture synchronization**		
Synchronized gesture	A gesture in synchrony with the object or activity that the children is attending to	The mother points to the car the child is playing with and says, “yellow car”.
Unsynchronized gesture	A gesture that is not synchronized with the object or activity the children is attending to	The mother points to the car the toddler is playing with and says, “yellow car”.

#### Toddler responses

**Figure 2:**
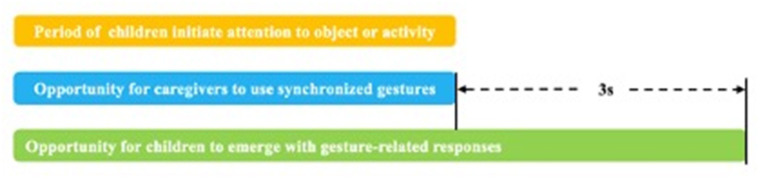
Synchronized gestures of the caregiver and gesture-related responses of the child. (1) After the child initiates attention to an object or activity in a dyadic activity (yellow bars), synchronized gestures in which the caregiver synchronizes with the toddlers' attention may occur during this period (blue bars). (2) The toddlers' gesture-related response to the caregiver's gesture was confirmed during the period from the time the gesture was presented until 3 s after the end of the gesture.

Toddler responses were coded within 3 s after the caregiver gesture was provided until the time the gesture ended (37). Referring to the concept of correlated response proposed by Kadlaskar et al. (38), responses were categorized as follows: no-response, where the toddler continued to perform activities after the caregivers' gestures appeared; gesture-related responses, where the toddler generated responses after the caregiver gesture was provided, including temporal overlap, where toddlers' responses appeared within 3 s after the gesture was provided, and content overlap, where the toddler developed a gesture-related response (Figure 2); and non-gesture-related responses, where the toddler developed a response after the caregiver gesture was provided, but not related to the communicative message intended by the gesture. We classified toddler responses into the following: gesture-related attentional disengaging (GAD), gesture-related gestures (GGs), gesture-related actions (GAs), gesture-related language (GL), gesture-related integrative responses (GIs) and non-GAD, non-GGs (NGGs), non-GAs (NGAs), non-GL, and non-GIs (see Supplementary Material for details of toddler response types).

### Coding reliability

After reliability had been established using training videos, two coders independently coded the videos and overlapped 20% of the randomly selected videos to calculate interrater reliability. Interrater reliability was calculated using intraclass correlation coefficient (ICC). The agreement for caregiver gesture types was 0.982 (range, 0.975–0.987), and the proportion agreement for toddler response and classification of response were as follows: toddler response [ICC = 0.982 (range, 0.967–0.990)], gesture-related response [ICC = 0.964 (range, 0.944–0.976)], and non-gesture-related response [ICC = 0.920 (range, 0.878–0.948)]. For any disagreements, the researchers watched segments for which there was any disagreement and reached a consensus decision, which was then used for analysis.

### Data analyses

To verify possible differences in the distribution of sample variable groups and caregiver gestures in different activities, *t*-tests or non-parametric tests were conducted for continuous variables and chi-squared tests were conducted for categorical variables. To further compare differences in children's social responses to caregiver gestures. Based on the 50th percentile of use of gestures by the two caregivers, we divided the CTI between the two caregivers and toddler in the same activity into Group I (caregivers who use gestures more than 50th percentile) and Group II (caregivers who used gestures <50th percentile). Finally, we performed a multiple linear regression analysis to enter the demographic characteristics, toddlers' abilities, and the caregivers' gesture variables into the model to determine the strength of the correlation between the caregivers' gestures and the toddlers' responses.

Since the purpose of this study was to explore the effects of gestures as early social information input to children and no significant differences were observed in the basic information of the two caregivers, the gestures of both caregivers were considered to be a uniform source of social information learning for the children. Therefore, we used the two caregivers' gestural means to compare caregivers' gestural input across activities without distinguishing and comparing the use of different caregivers' gestures in the same activity. We corrected for all Bonferroni *p*-values.

## Results

Finally, 73 families (ASD = 44, TD = 29) completed the video of play activities; 61 families (ASD = 34, TD = 27) completed the videos of dining activities. The two groups did not differ in toddlers' physiological age, sex, caregiver age, caregiving time, education, or household income. We also compared the two groups of caregivers who were both caregivers, and no significant differences were found (play: χ^2^ = 0.920, *p* = 0.337; dining: χ^2^ = 0.397, *p* = 0.529). According to Mullen's definition of developmental delay (T scores of <30), ASD showed significant delay in terms of language abilities, including receptive language (RL) (play: χ^2^ = 18.156, *p* < 0.01, df = 1; dining: χ_2_ = 15.796, *p* < 0.01, df = 1) and EL (play: χ^2^ = 29.937, *p* < 0.01, df = 1; dining: χ^2^ = 25.432, *p* < 0.01, df = 1). In terms of non-verbal abilities, among toddlers with ASD who participated in play and meal activities, 8 and 6 had VR delays and 4 and 3 had FM delays, respectively. And most toddlers in both groups had non-verbal abilities at normal levels, including visual reception (VR) (play: χ^2^ = 5.922, *p* = 0.015, df = 1; dining: χ^2^ = 5.284, *p* = 0.022, df = 1) and fine motor (FM) (play: χ^2^ = 2.789, *p* = 0.095, df = 1; dining: χ^2^ = 2.506, *p* = 0.113, df = 1). Toddler with ASD scored higher the calibrated severity score (CSS) of the ADOS-T than TD (Table 1).

### Caregiver gesture

To solve the first question, we compared the frequencies of the total number of caregiver gestures, different gesture communication function types, gesture-speech combinations types, and gesture synchronization in CTI. As shown in Table 3, caregivers of toddlers with ASD and TD produced a similar total frequency of gestures during play (*Z* = −0.051, *p* = 0.959, df = 71) and dining (*Z* = −1.456, *p* = 0.146, df = 59) activities. For speech-gesture combinations, there was no significant difference in reinforcing and disambiguating gestures between the two groups (Bonferroni, *p* > 0.005). However, notably, the frequency of supplementary gestures in the ASD group was higher than that in the TD group during dining activity (*Z* = −2.895, *p* = 0.004, df = 59). During play activity, significant differences were observed between the two groups regarding synchronized gestures (*t* = −4.407, Bonferroni, *p* < 0.005, df = 71) and unsynchronized gestures (*Z* = −3.642, Bonferroni, *p* < 0.005, df = 71), indicating fewer synchronized gestures but more unsynchronized gestures from caregivers of children with ASD than TD. However, during dining activity, there were no significant differences regarding these two types of gestures in either of the two activities. This indicated that different types of gestures were used by the caregiver in different activities.

**Table 3:** Gesture frequency of caregivers caring for toddlers with autism spectrum disorder (ASD) and typically developing (TD) toddlers during different activities.

**Caregiver gestures types** ***M*** **(SD)**	**Play activity**		**Dining activity**	
	**ASD (*****n*** **= 44)**	**TD (*****n*** **= 29)**	**ASD (*****n*** **= 34)**	**TD (*****n*** **= 27)**
Total gestures	24.14 (8.25)	23.62 (8.13)	11.44 (7.08)	8.56 (3.88)
Communicative function
Behavior regulation	11.02 (5.11)	11.79 (5.74)	3.97 (3.16)	4.04 (2.10)
Social interaction	0.93 (1.28)	0.93 (0.96)	1.00 (1.54)	0.85 (1.32)
Joint attention	12.55 (5.51)	11.34 (4.64)	5.17 (4.99)	4.00 (2.81)
Gesture-speech combination
Reinforcing gestures	7.48 (4.83)	6.21 (3.79)	4.09 (3.23)	3.70 (3.23)
Disambiguating gestures	5.55 (3.33)	6.41 (3.77)	1.76 (2.12)	1.70 (1.35)
Supplementary gestures	10.39 (4.38)	9.66 (4.75)	5.41 (3.82)	2.78 (1.25)^**#**^
Gesture synchronization
Synchronized gesture	8.84 (5.38)	14.14 (4.42)^**#**^	6.29 (3.75)	5.11 (3.47)
Unsynchronized gesture	15.48 (6.86)	9.73 (5.84)^**#**^	5.41 (4.44)	3.67 (2.47)

*Data are reported as group means with standard deviations in parentheses. ^**#**^Adjusted p < 0.005*.

### Toddler response

#### Response proportion in different activities for the two groups

**Figure 3:**
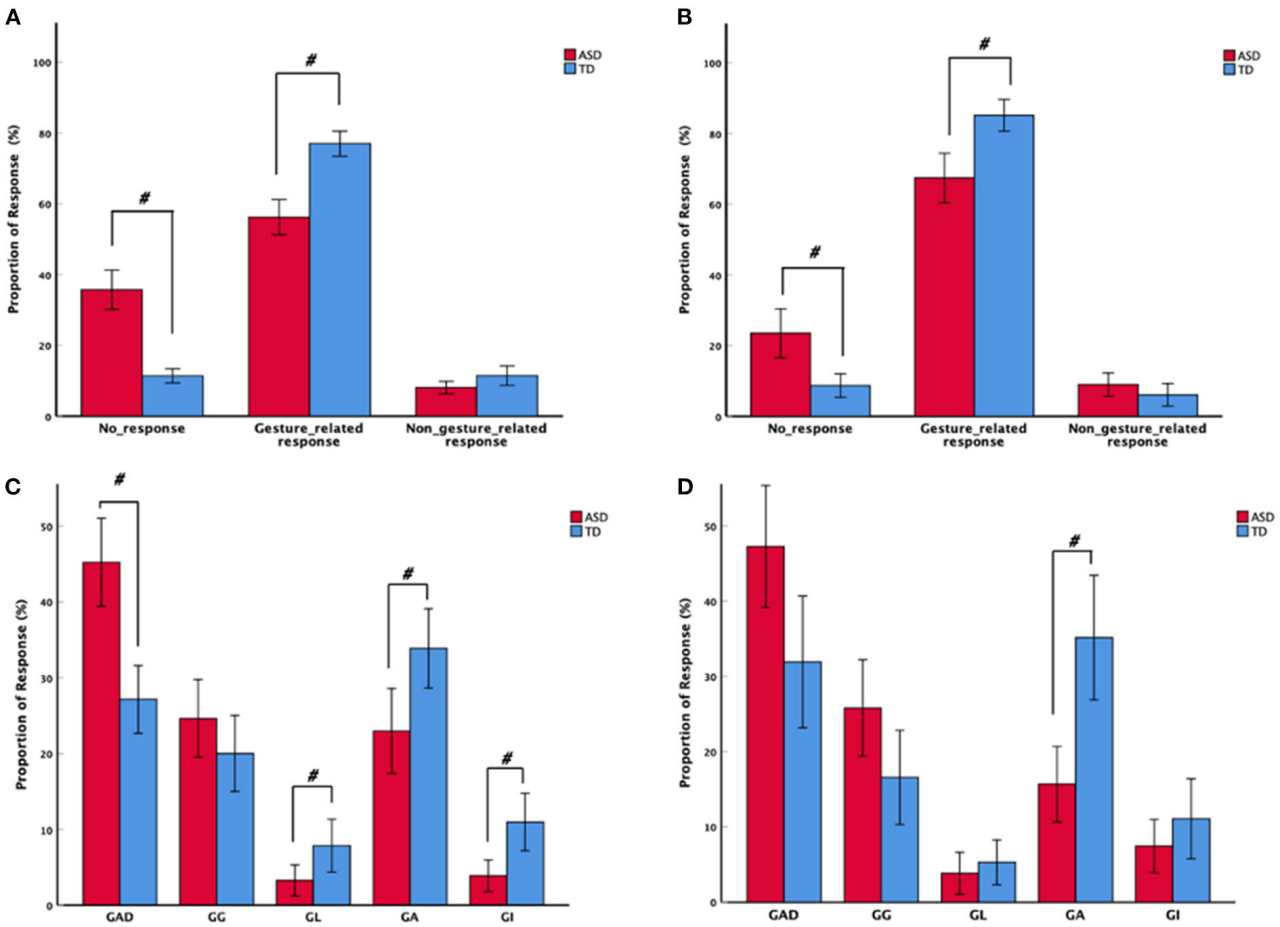
Mean proportion of toddler response types to caregivers' gestures among the autism spectrum disorder and typically developing groups in **(A)** play and **(B)** dining activities. Mean proportion of toddler gesture-related responses, including gesture-related attentional disengaging, gesture-related gestures, gesture-related actions, gesture-related language, and gesture-related integrative responses, to caregivers' gestures in **(C)** play and **(D)** dining activities. With error bars showing ± 2 standard error. ^#^Adjusted *p* < 0.005.

We first explored whether the proportion of no-responses, gesture-related responses, and non-gesture-related responses categories differed between the two groups. There were significant group differences in proportion of no-responses and gesture-related responses, indicating that toddlers with ASD had a significantly greater proportion of no-responses (play activity: *t* = 4.502, Bonferroni, *p* < 0.005, df = 71; dining activity: *Z* = −3.277, Bonferroni, *p* < 0.005, df = 59) and lower gesture-related responses to caregiver gestures compared to TD toddlers (play activity: *t* = −6.122, Bonferroni, *p* < 0.005, df = 71; dining activity: *t* = −4.033, Bonferroni, *p* < 0.005, df = 59) (Figure 3). However, there were no significant differences in the proportion of non-gesture-related responses (play activity: *Z* = −1.809, *p* = 0.071, df = 71; dining activity: *Z* = −1.341, *p* = 0.180, df = 59) between the two groups. This suggests that toddlers in the ASD group were less responsive to caregiver gestures compared to TD toddlers when they were engaged in CTI.

We next examined whether the proportion of each gesture-related response type (GAD, GGs, GL, GAs, GIs) differed between the two groups. Results indicated a significant difference in the proportion of GAD, GL, GA, and GI responses across the two groups, indicating that toddlers with ASD displayed significantly more GAD (*Z*= −3.861, Bonferroni, *p* < 0.005, df = 71) and fewer GL (*Z* = −2.982, *p* = 0.003, df = 71), GAs (*Z* = −3.079, *p* = 0.002, df = 71), and GIs (*Z* = −3.083, *p* = 0.002, df = 71) compared to TD toddlers during play activity. However, during dining activity, the ASD group produced significantly fewer GAs compared to the TD group (*Z* = −3.594, Bonferroni, *p* < 0.005, df = 59). This suggests that toddlers with ASD respond differently to caregiver gestures in different activities. Further, toddlers with ASD mainly showed attentional disengaging and gesture responses to caregiver gestures, whereas TD toddlers showed more language, action, and integrative responses than toddlers with ASD.

#### Respose proportion to different frequencies of gesture use in activities

**Figure 4:**
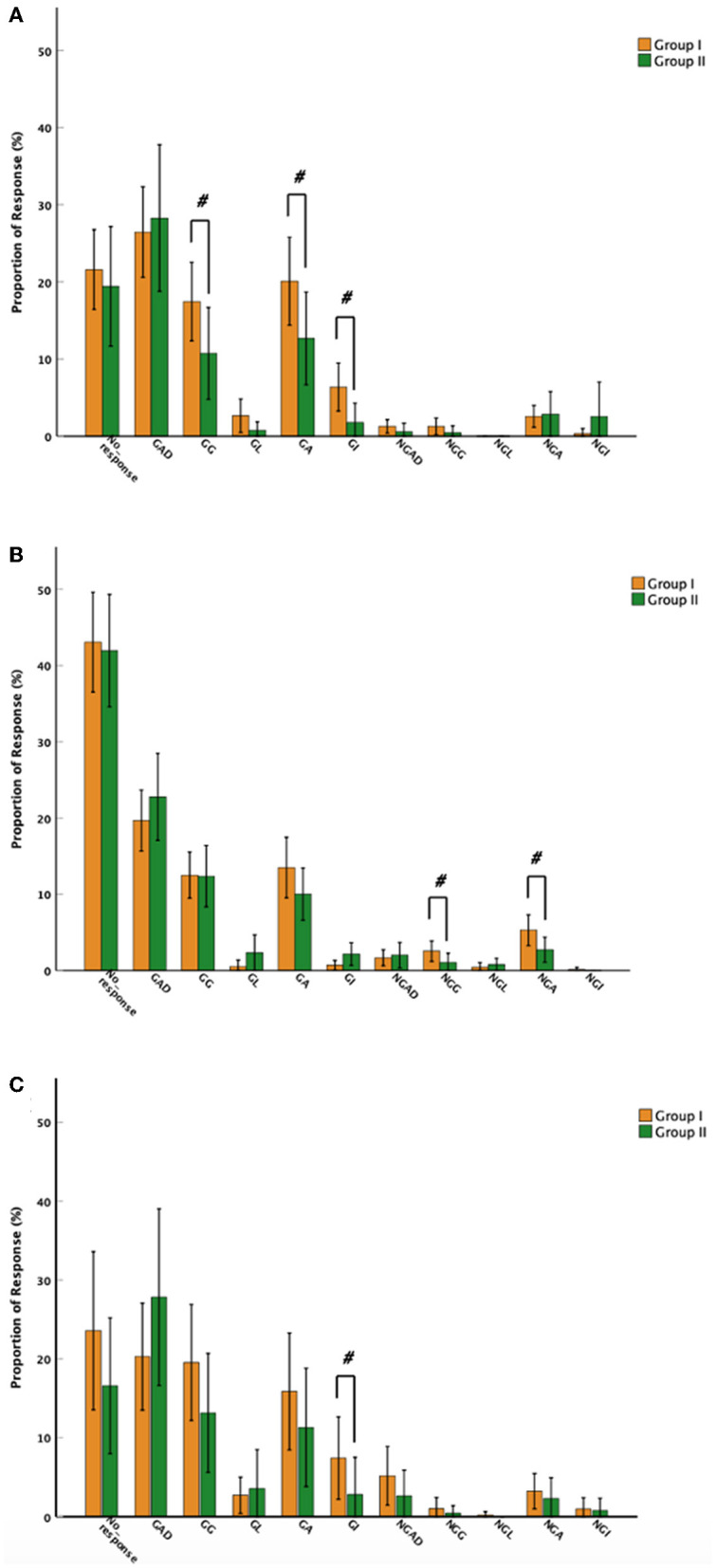
Mean proportion of toddler response types to caregivers' gestures among groups I and II. **(A)** Toddlers with autism spectrum disorder (ASD) responses to synchronized gestures in play activities; **(B)** ASD responses to unsynchronized gestures in play activities. **(C)** Typically developing toddlers' responses to supplementary gestures in dining activities. Toddler responses include toddler responses into the following: gesture-related attentional disengaging (GAD), gesture-related gestures (GGs), gesture-related actions (GAs), gesture-related language (GL), gesture-related integrative responses (GIs) and non-GAD, non-GGs, non-GAs (NGAs), non-GL (NGL), and non-GIs, with error bars showing ± standard error. ^#^Adjusted *p* < 0.005.

The above results suggested that toddlers with ASD may respond differently to synchronized and unsynchronized gestures from caregivers during play activities, and that TD toddlers may respond differently to supplementary gestures during dining activities. We therefore further analyzed the children's responses to the three gestures during two activities. Groupings were made according to the frequency of the two caregivers' gestures used to explore the differences in toddlers' social responses to caregiver gestures during two activities in both the ASD and TD groups. Based on the 50th percentile of use of gestures by the two caregivers, two caregivers and toddler in the same activity into group I (n_synchronized_ = 43, n_unsynchronized_ = 39, n_supplementary_ = 25) and group II (n_synchronized_ = 45, n_unsynchronized_ = 49, n_supplementary_ = 43). Toddlers with ASD produced a significantly greater proportion of GGs (*Z* = −2.879, *p* = 0.004, df = 86), GAs (*Z* = −2.856, *p* = 0.004, df = 86), and GIs (*Z* = −3.947, Bonferroni, *p* < 0.005, df = 86) in synchronized gestures group I than those in group II during play activity, whereas the unsynchronized gestures in group I had significantly more NGGs (*Z* = −3.634, *p* < 0.005, df = 86) and NGAs (*Z* = −2.869, *p* = 0.004, df = 86) than group II. In dining activities, TD toddlers in supplementary gesture group I had significantly more GIs (*Z* = −2.973, *p* = 0.003, df = 66) than group II (Figure 4).

Overall, caregivers of toddlers with ASD who used synchronized gestures during play activities produced more gesture-related responses (GGs, GAs, GIs) and increased non-gesture-related responses (NGGs, NGAs) with unsynchronized gestures. In addition, caregivers of TD toddlers produced more GIs using supplementary gestures during dining activities.

#### Correlation between toddler response and caregiver gesture

**Figure 5:**
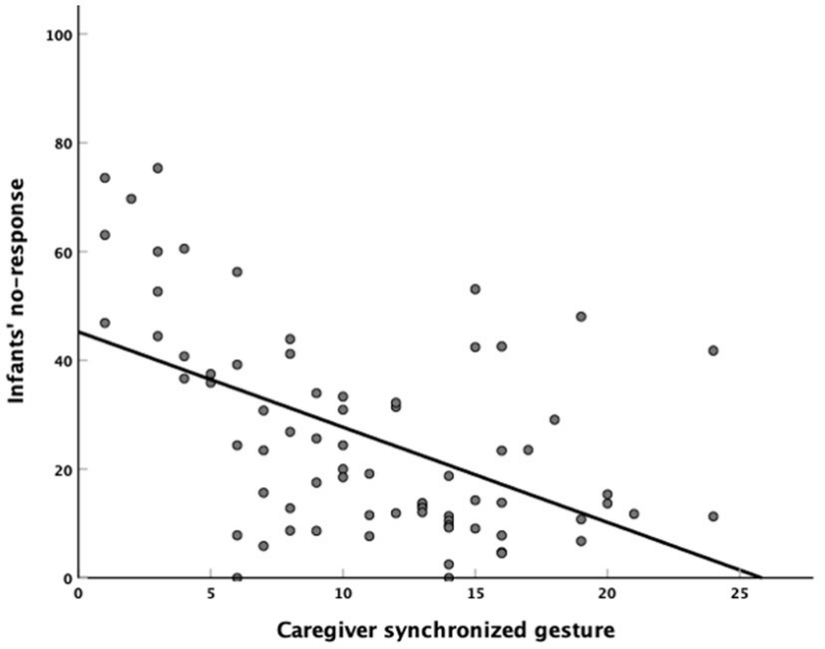
Scatterplot with a best-fit line depicting the relationship between caregiver's synchronized gestures and autism spectrum disorder's no-responses during play activity.

We combined the differences in caregiver gestures and toddler responses to caregiver gestures between the two groups. To examine the relationship between caregiver gestures and toddler responses during CTI, synchronized and asynchronized gestures of the caregivers during play activities were entered into a series of three regression models predicting toddlers' social responses in CTI. The caregivers' synchronized gestures were entered into a series of three regression models predicting toddler's no-response in play activities. Model 1 included demographic information parameters (toddler's age, sex). Model 2 included toddler parameters (non-verbal score, verbal score, and ADOS CSS). The final model 3 included caregiver synchronized gestures. Compared to models 1 and 2, model 3 (the addition of caregiver synchronized gestures: β = −0.708, *t* = −2.505, *p* = 0.015, df = 1) accounted for a significantly greater portion of toddlers' no-response variance (*R*^2^ change = 0.034, F change = 6.277, *p* = 0.015, df = 71, Figure 5). Similar to no-response, model 3 applied to the GA and GI responses in play activities did not reveal significance with synchronized gestures (*p* > 0.015). The results of the regressions indicated that caregiver synchronized gestures predicted no-response of ASD during play activity.

## Discussion

We examined the characteristics of caregiver gestures and toddlers' responses to caregiver gestures in early family environments and the relationship between caregiver gestures and toddlers' social responses. Our results indicate that caregivers of toddlers with ASD used different types of gestures in different activities, whereas children with ASD had different types of responses to caregivers in both activities. In addition, caregivers used synchronized gestures during play and supplementary gestures during dining, which increased the gesture-related responses exhibited by toddlers with ASD. Finally, for ASD CTI, synchronized gestures by the playful caregiver effectively predicted toddlers' social responses. We discuss possible explanations for the pattern of caregiver gesture use as a key behavior in early CTI.

First, we observed that the overall frequency of gesture use was similar between the two groups, consistent with previous studies that reported ASD and TD caregivers use a similar number of gestures (11, 39). We also observed that caregivers of toddlers with ASD used fewer synchronized gestures and more unsynchronized gestures during play activities and more supplementary gestures during dining activities. This extends previous evidence on caregiver gesture types into a new domain, as previous reports on caregiver gestures were recorded only in play settings and mostly involved gesture patterns (7, 8). In this study, multi-scenario and different gesture type reports were relevant. We observed that the use of unsynchronized gestures by caregivers of toddlers with ASD in games was mostly used to instruct toddlers to pay attention to and recognize novel items. Relatively consistent with this result is a study on parenting instructional styles, which reported that in situations where toddlers have fewer functional games and behaviors, caregivers of toddlers with ASD dominate more interaction and do not make the dyad more mutual (40, 41). Therefore, the use of unsynchronized gestures in caregivers of toddlers with ASD may not be beneficial.

Supplementary gestures during dining are mostly used to further add clarification to toddlers' concerns and are a more complex pattern of gesture-speech combinations than reinforcing and disambiguating gestures. Most studies report no group differences in the number of gesture-verbal combinations provided to toddlers by their caregivers during play activities (7, 13). In contrast, this study's reporting of supplementary gestures in everyday situations (dining activities) may reveal caregivers' verbal fine-tuning, according to their toddlers' communicative needs in different situations. The present findings revealed group differences in caregiver gestures across contexts that may reflect the specific effects of caregiver gestures on early CTI.

Second, in gesture-related responses, ASD is associated with more attentional disengaging and less language, actions, and integrative responses, reflecting a marked lag in the development of social skills for this age group (28, 42). Although different from previous assessments of children's competence development, observations of children's responses to caregiver gestures may serve as a way to gain initial insight into the early social skill levels of children with ASD.

Third, although the poor response to gestures by toddlers with ASD is associated with a lack of interest in social stimuli (43, 44), our study provides evidence for the possibility of increasing social responses in ASD through effective use of caregiver gestures. In the context of low CTI response in the early stages of ASD (41, 45), we observed that the use of synchronized gestures by the caregiver increased gesture-related responses in toddlers with ASD, whereas unsynchronized gestures increased gesture-unrelated responses, suggesting that using synchronized gestures may be a key behavior in promoting toddlers' engagement in CTI. We also observed that caregivers of toddlers with ASD use synchronized gestures mostly to indicate and demonstrate to toddlers how to manipulate objects during play and to respond to toddlers' needs in a timely manner. This suggests that carefully following toddlers' interests during CTI and responding to their communication behaviors in a timely manner are effective ways to provide synchronized gestures to toddlers with ASD. Evidence related to this result included a report on synchronous behaviors of parenting to promote joint attention and engagement in ASD that indicated that parental responses to toddlers' attention and activities during play was positive for toddler initiations with caregiver and for caregiver-toddler shared attention (21, 46). Therefore, the study of synchronized gestures provides specific ways for caregivers to increase synchronous behavior in children.

Finally, by examining basic toddler information developmental and symptom levels, we observed that synchronized caregiver gestures were associated with no response in interaction with ASD, suggesting that more synchronized gestures by the caregiver may be associated with reduced non-responsiveness from children.

Although previous studies have addressed the effects of synchronous caregiver behavior on children's developmental abilities and disorder symptoms (25, 47), our study provides further preliminary scientific evidence for the use and positive effects of caregiver synchronized gestures in natural family settings.

### Limitations

As a cross-sectional study, the present study highlights group differences between key developmental transition points and cannot reveal trajectories that deviate from typical milestones and cannot account for causal relationships between behaviors. Also, although there were no significant differences between the two groups of children's non-verbal abilities in this study, 18–24 months of age is an important stage in children's ability development, and the impact of changes in the developmental characteristics of children's abilities, both verbal and non-verbal (gestures), on caregiver-toddlers interaction and children's social behavior during this stage was not considered in this study. Further, due to sample size limitations and the language ability differences among the two groups, the current study cannot exclude that children's social responses in CTI are a manifestation of early competence developmental limitations. Thus, the effect of caregiver gestures on children's social responses in early childhood needs to be further illustrated by a prospective cohort design. Finally, the current results are aimed at CTI with young children and cannot be extended to older children and other contexts.

## Conclusion

In summary, these findings reveal that the specificity of caregiver gesture use in early CTI for ASD is demonstrated by synchronized gestures during play and supplementary gestures during dining activities, whereas synchronized gestures by the caregiver during play contribute to the development of social responses and competence in CTI for ASD. Coaching caregivers on how to use gestures more effectively can promote joint participation in CTI for children with ASD. Thus, the relationship between caregiver gestures and children's responses in early CTI warrants further studies, as it may be a moderator of children's social development and intervention effects.

## Data Availability Statement

The raw data supporting the conclusions of this article will be made available by the authors, without undue reservation.

## Ethics Statement

The studies involving human participants were reviewed and approved by Medical Ethics Committee of the Third Affiliated Hospital of Sun Yat-sen University. Written informed consent to participate in this study was provided by the participants' legal guardian/next of kin.

## Author contributions

SL, XZ, and HD conceived the current study and contributed to the interpretation of the results. SL, YX, and CW participated in data collection. SL, HZ, and LL performed the analyses. SL took the lead in writing the manuscript. HD and LL supervised the writing. All authors provided critical feedback and helped shape the research, analysis, and manuscript.

## Funding

This work was supported by the Science and Technology Program of Guangzhou, China, Key Area Research and Development Program (202007030011).

## Conflict of interest

The authors declare that the research was conducted in the absence of any commercial or financial relationships that could be construed as a potential conflict of interest.

## Publisher's note

All claims expressed in this article are solely those of the authors and do not necessarily represent those of their affiliated organizations, or those of the publisher, the editors and the reviewers. Any product that may be evaluated in this article, or claim that may be made by its manufacturer, is not guaranteed or endorsed by the publisher.
