# Supplementary material for: The role of caregiver gestures and gesture-related responses of toddlers with autism spectrum disorder
Source: Front Psychiatry. 2022 Jul 22;13:895029. doi: 10.3389/fpsyt.2022.895029 (PMC9353329; doi:10.3389/fpsyt.2022.895029)
Supplement: Supplementary file 1 [file Table_1.DOCX]

Supplementary Material

# Supplementary Table

**Infant response types Definition**

| **Infant responses** | **Definition** | **Example** |
| --- | --- | --- |
| **attentional disengaging** | The child's attention to the object or activity from which the caregiver's gesture emanates is demonstrated by shifting the eye away from the current focus to something related to the nurturer's gesture. | The caregiver points to the blocks and says, "Look," and the child looks at the blocks. |
| **gestures** | Gestures produced by the child after the gesture of the caregiver. | The caregiver waves goodbye to the child and the child waves goodbye. |
| **actions** | Through various body movements, thus replacing words to express the purpose of communication, including body and limb communication movements other than hand gestures. | The caregiver tells the child, "Sit in the chair," and the child follows the instruction to sit in the chair. |
| **language** | A system of instructions that consists of vocabulary and/or grammar and expresses ideas. | The caregiver hands a toy to a child and the child says, "Do not play with this" |
| **integrative responses** | Two or more of the above three reaction types occur simultaneously. | The caregiver holds up the blocks and asks, "What color is this?" and the child points to the blocks and correctly, verbally identifies the color. |
